# Sex- and age-specific trends in antibiotic resistance patterns of *Escherichia coli* urinary isolates from outpatients

**DOI:** 10.1186/1471-2296-14-25

**Published:** 2013-02-22

**Authors:** Jessina C McGregor, Miriam R Elman, David T Bearden, David H Smith

**Affiliations:** 1 Department of Pharmacy Practice, College of Pharmacy, Oregon State University/Oregon Health & Science University, 3303 SW Bond Ave., CH12C, Portland, OR, 97239, USA; 2 Kaiser Permanente Northwest Center for Health Research, Portland, OR, USA

**Keywords:** Urinary tract infection, Urinary anti-infectives, Escherichia coli

## Abstract

**Background:**

Urinary tract infections (UTIs) are one of the most common infections treated in ambulatory care settings, however the epidemiology differs by age and sex. The incidence of UTI is far greater in females than males, and infection in pediatric patients is more often due to anatomical abnormalities. The purpose of this research was to describe age- and sex-specific trends in antibiotic susceptibility to common urinary anti-infectives among urinary isolates of *Escherichia coli* from ambulatory primary care patients in a regional health maintenance organization.

**Methods:**

Clinical microbiology data were collected for all urine cultures from patients with visits to primary care clinics in a regional health maintenance organization between 2005 and 2010. The first positive culture for *E. coli* tested for antibiotic susceptibilities per patient per year was included in the analysis dataset. The frequency of susceptibility to ampicillin, amoxicillin-clavulanate, ciprofloxacin, nitrofurantoin, and trimethoprim/sulfamethoxazole (TMP/SMX) was calculated for male and female patients. The Cochrane-Mantel-Haenzel test was used to test for differences in age-stratified susceptibility to each antibiotic between males and females.

**Results:**

A total of 43,493 *E. coli* isolates from 34,539 unique patients were identified for study inclusion. After stratifying by age, *E. coli* susceptibility to ampicillin, amoxicillin-clavulanate, ciprofloxacin, and nitrofurantoin differed significantly between males and females. However, the magnitude of the differences was less than 10% for all strata except amoxicillin-clavulanate susceptibility in *E. coli* isolated from males age 18–64 compared to females of the same age.

**Conclusions:**

We did not observe clinically meaningful differences in antibiotic susceptibility to common urinary anti-infectives among *E. coli* isolated from males versus females. These data suggest that male sex alone should not be used as an indication for empiric use of second-line broad-spectrum antibiotic agents for the treatment of UTIs.

## Introduction

Urinary tract infections (UTIs) are one of the most commonly treated bacterial infections and account for over 10 million ambulatory care visits annually in the United States [1]. Antibiotic treatment is typically selected empirically, based on the patient clinical presentation, medical history and local patterns of antibiotic susceptibility [2]. Because the incidence of UTIs is significantly greater among women, much of the research has focused on women. Thus there exists a paucity of research on UTIs in men. As such, guidelines on the diagnosis, treatment, and management of UTIs focus largely on infection in women [2-5].

The incidence, presentation, and course of infection for UTIs in men and women differ in large part due to anatomical differences. For the treatment of acute uncomplicated cystitis in women, trimethoprim/sulfamethoxazole (TMP/SMX), nitrofurantoin, fosfomycin, and pivmecillinam are recommended first-line empiric therapies [2,6]. In men, because prostate involvement occurs in roughly 90% of cases, an empiric agent should be selected that achieves therapeutic concentrations in prostatic tissues (e.g., trimethoprim or ciprofloxacin) [7,8].

While historically it was believed that the causative organism in UTIs differed between men and women, more recent data has shown that for both sexes the primary causative pathogen is *Escherichia coli*, which accounts for 75-90% of UTIs [8,9]. With the increases in antibiotic resistance among *E. coli* and other *Enterobacteriaceae* over the past several decades, surveillance data have become critical for appropriate empiric selection of antibiotic therapy. U.S. guidelines specify that TMP/SMX should be avoided for empiric treatment of uncomplicated acute cystitis or pyelonephritis in populations where non-susceptibility to this agent exceeds 20% in uropathogens [2].

While some surveillance studies have identified significant differences in the frequency of susceptibility to common urinary anti-infectives between isolates collected from male and female patients, this has not been consistently observed [10,11]. The objective of this study was to describe age- and sex-specific antibiotic susceptibility patterns for common urinary anti-infectives among *E. coli* urine isolates using six years of data from a large ambulatory primary care patient population.

## Methods

### Study design and patient population

We conducted a cross-sectional study of urinary *E. coli* isolates from outpatients of Kaiser Permanente Northwest (KPNW) primary care clinics. KPNW is a regional health maintenance organization that serves over 485,000 members in northwest Oregon and southwest Washington. Primary care clinics were defined as non-specialty care clinics within the Family Practice, Internal Medicine, or Pediatrics departments; all primary care clinics were included. Urine cultures positive for *E. coli* that were drawn from patients with visits in the primary care clinics between January 1, 2005 and December 31, 2010 were eligible for inclusion in the analysis. Cultures in which less than 10,000 colonies/mL were identified or 3 or more organisms were isolated were excluded. The analysis dataset was then limited to the first isolate tested for antibiotic susceptibilities per patient and year to minimize potential bias resulting from repeat culturing [12].

### Data collection

Data were electronically extracted from the virtual data warehouse maintained by the Kaiser Permanente Center for Health Research. Collected data included patient demographics, department in which the clinic visit occurred, and all clinical microbiology data for urine cultures. Approval for this study was obtained from the KPNW institutional review board.

### Data analysis

The frequency of susceptibility to ampicillin, amoxicillin-clavulanate, ciprofloxacin, nitrofurantoin, and trimethoprim/sulfamethoxazole (TMP/SMX) was calculated for *E. coli* isolates stratified by patient sex and year. The Cochrane-Armitage test for trend was used to identify changes in the frequency of susceptibility to each antibiotic over time independently among males and females. Patient age at the time of culture was categorized as less than 18 years, 18–64 years, and 65 years and older. The frequency of susceptibility to each antibiotic was also compared across age categories among males and females using the Cochran-Mantel-Haenszel test. An alpha level less than or equal to 0.05 was the statistical significance level for all analyses and data were analyzed with SAS (version 9.2, SAS Corporation). Clinically significant differences were defined as differences of 10% or greater.

## Results

### Description of study sample

During the study time frame 190,396 urine cultures were performed at primary care clinic visits; 70,180 were positive for *E. coli* and 57,550 of those were tested for antibiotic susceptibilities. After restricting the data to the first isolate per patient per year, 43,493 isolates from 34,539 unique patients remained in the final analysis data set. Table 1 describes the demographics of these patients. Of the included isolates, 2,520 (5.8%) were from male patients.

**Table 1:** **Patient characteristics based on first *E. coli *isolate from urine specimen in KPNW outpatients 2005-2010**^**a**^

**Characteristic**	**n = 34539**	
Mean age (SD)	47.0 (22.4)	
Female	32265	(93.4)
Ethnicity		
Hispanic	1067	(3.1)
Non-Hispanic	16984	(49.2)
Unknown/missing	16488	(47.7)
Race		
White	20974	(60.7)
Black	377	(1.1)
Native American/Alaskan Native	108	(0.3)
Asian	1411	(4.1)
Pacific Islander/Hawaiian	46	(0.1)
Other	767	(2.2)
Unknown	10856	(31.4)

^a^Data are No. (%) unless otherwise indicated.

### Antibiotic susceptibility of urinary Escherichia coli isolates by patient sex

Overall, 66.0% (1,664/2,520) of *E. coli* isolated from males and 66.3% (27,175/40,971) from females were susceptible to ampicillin. Amoxicillin-clavulanate susceptibility was 56.9% (484/850) among males and 67.3% (9,291/13,798) among females; however, it should be noted that testing for susceptibility to amoxicillin-clavulanate was routinely performed and reported only for ampicillin non-susceptible *E. coli*. Ciprofloxacin susceptibility for the *E. coli* isolates was 93.2% (2,292/2,458) among males and 95.9% (37,900/39,524) among females, and nitrofurantoin susceptibility was 96.4% (2,430/2,520) among males and 97.6% (39,979/40,968) among females. For TMP/SMX, 86.3% (2,176/2,520) of *E. coli* isolated from males were susceptible compared to 84.7% (34,674/40,958) among females.

### Trends in antibiotic susceptibility of urinary Escherichia coli isolates over time

**Figure 1:**
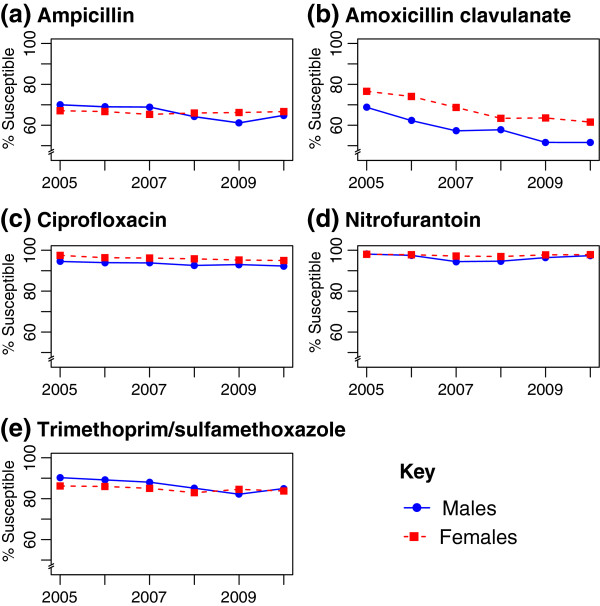
**Susceptibility to urinary anti-Infectives by sex and year for *E. coli *cultured from urine.** For all figures, males shown as blue solid line, females as red dashed line. **a**, ampicillin susceptibility (test for trend: males, p=0.058; females, p=0.6256); **b**, amoxicillin clavulanate susceptibility (test for trend: males, p=0.0006; females, p<0.0001); **c**, ciprofloxacin susceptibility (test for trend: males, p=0.1445; females, p<0.0001); **d**, nitrofurantoin susceptibility (test for trend: males, p=0.4967; females, p=0.5508); **e**, trimethoprim/sulfamethoxazole susceptibility (test for trend: males, p=0.0004; females, p<0.0001).

Figure 1 presents the susceptibility of *E. coli* to each antibiotic by sex and year. Susceptibility to amoxicillin-clavulanate and TMP/SMX decreased significantly over time among both males and females. Ciprofloxacin susceptibility also decreased significantly over time among females, but not males. Table 2 presents the age stratified antibiotic susceptibilities for *E. coli* isolated from males and females. The age-specific susceptibilities differed significantly between males and females for all antibiotics except TMP/SMX.

**Table 2:** Susceptibility to urinary anti-infectives by sex and age for *E. coli *cultured from urine*

	**Age (years)**			**CMH**
	**<18**	**18-64**	**>64**	**p-value**
Ampicillin				<0.0001
Male	104 (58.8)	852 (68.5)	708 (64.4)	
Female	2564 (62.6)	16988 (65.4)	7623 (69.9)	
Amoxicillin clavulanate				<0.0001
Male	45 (64.3)	214 (55.6)	225 (57.0)	
Female	1113 (73.1)	6040 (67.5)	2138 (64.2)	
Ciprofloxacin				<0.0001
Male	112 (97.4)	1201 (96.5)	979 (89.1)	
Female	2613 (98.4)	25161 (96.9)	10126 (92.8)	
Nitrofurantoin				<0.0001
Male	174 (98.3)	1214 (97.6)	1042 (94.8)	
Female	4023 (98.2)	25413 (97.9)	10543 (96.6)	
Trimethoprim/Sulfamethoxazole				0.3888
Male	147 (83.1)	1090 (87.6)	939 (85.4)	
Female	3479 (84.9)	21909 (84.4)	9286 (85.1)	

*Represents the first isolate for each patient and year.

*CMH:* Cochrane Maentel Haenszel correlation test.

## Discussion

In this study, we observed statistically significant differences between males and females in the age-specific susceptibilities of *E. coli* to ampicillin, amoxicillin-clavulanate, ciprofloxacin, and nitrofurantoin. Urinary *E. coli* isolates from male patients tended to exhibit increased antibiotic resistance than isolates from female patients. Despite the statistical significance of time trends and differences in age-specific susceptibilities, the magnitude of these differences was generally less than 5% and thus may not represent clinically meaningful differences. The exception was susceptibility to amoxicillin-clavulanate, where susceptibility was roughly 10% lower in males age 18 to 64 years than females in the same age group. Yet these differences should be interpreted cautiously because susceptibility to amoxicillin-clavulanate was only provided for ampicillin non-susceptible *E. coli* isolates in the current analysis. If all ampicillin susceptible isolates are assumed to be amoxicillin-clavulanate susceptible, then the magnitude of the differences between males and females would be less than 5% in all age categories (difference not statistically significant).

While few other studies have explored the differences in antibiotic susceptibility of uropathogens isolated from male and female ambulatory patients, our findings are consistent with the trends observed in the literature. A recent 10-year study of community UTI in Portuguese patients also identified differences in antibiotic susceptibility by patient sex. The authors reported that urinary isolates of *E. coli* were significantly more resistant to fluoroquinolones, penicillins, nitrofurantoin, and first and second generation cephalosporins among men compared to women [13]. Another study focused on pediatric patients, also identified significantly higher resistance to TMP/SMX and ciprofloxacin in male versus female patients [14]. The NAUTICA surveillance study of outpatient UTIs reported greater antibiotic resistance to ciprofloxacin, levofloxacin, and TMP/SMX among all urinary isolates from U.S. and Canadian male patients [15]. In the CANWARD study, antibiotic susceptibility among all *E. coli* isolates (not limited to urine isolates) collected from Canadian tertiary medical centers were compared and resistance was also observed to be significantly higher to ciprofloxacin, levofloxacin, and TMP/SMX in isolates collected from male patients versus female patients [11].

The prevalence and susceptibilities of antibiotic-resistant bacteria varies widely by geographic region, thus these data may not be generalizable to all regions. Local surveillance data of antibiotic susceptibilities would be needed to validate these findings in different patient populations. It should be noted that in our study resistance to TMP/SMX did not exceed 20%, as it does in many other regions of the U.S., thus in this patient setting TMP/SMX may be a viable empiric treatment option in both males and female patients. Additionally, antibiotic susceptibilities may differ by other patient or infection characteristics (e.g., type of UTI). These data were not available for analysis in this study and further investigation in this area is needed. Also, because clinical signs and symptoms of infection were not available and because not all suspected infections are cultured, the use of all urinary microbiology from this population cultures may not represent all true infections.

Currently there exists insufficient data to inform the development of evidence-based guidelines for the treatment of community UTIs in men. UTIs in adult males are considered complicated infections according to most clinical definitions [16-18]. Treatment recommendations vary from longer durations of therapy to the selection of broad-spectrum agents such as fluoroquinolones [6]. These recommendations are driven by the high proportion of UTIs in men with prostate involvement and concerns surrounding antibiotic resistance to TMP/SMX [7,8]. Previous research among Swiss outpatients has demonstrated the selection of fluoroquinolones versus TMP/SMX for the treatment of UTI is often influenced by nonclinical factors [19]. In this study, TMP/SMX, a recommended first-line agent for UTIs in both children and adults, the differences in susceptibility between *E. coli* isolated from males and females were neither statistically nor clinically significant and resistance rates are below 20%. Consequently, in this population, there is no evidence that male sex alone should be an indication for empiric selection of a second-line broad-spectrum antibiotic agent for the treatment of UTI. While more research is needed on treatment effectiveness of different regimens used to treat community UTIs in men, population-specific antibiotic susceptibility data are a necessary component in the empiric antibiotic selection process.

## Conclusions

In the era of increasing antibiotic resistance, prudent use of antibiotics is critical to prolong the clinical effectiveness of existing agents. Excessive use of broad-spectrum agents, such as fluoroquinolones, increases the evolutionary selective pressures that drive the increasing prevalence of resistance. These data suggest that first-line urinary anti-infectives such as TMP/SMX may be effective agents for treating UTIs in men. While more data are needed, clinicians should use local surveillance data to guide the prudent, empiric selection of antibiotic therapy for UTIs.

## Abbreviations

UTI: Urinary Tract Infection; KPNW: Kaiser Permanente Northwest; TMP/SMX: trimethoprim/sulfamethoxazole

## Competing interests

The authors declare that they have no competing interests.

## Authors’ contributions

JCM participated in the conception and design of the study; the acquisition, statistical analysis, and interpretation of the data; and the drafting of the manuscript. JCM, MRE, DTB, and DHS assisted with the interpretation of the data and participated in the critical revision of the manuscript. All authors read and approved the final manuscript.

## Pre-publication history

The pre-publication history for this paper can be accessed here:

http://www.biomedcentral.com/1471-2296/14/25/prepub

## References

[B1] Schappert SM Rechtsteiner EA Ambulatory medical care utilization estimates for 2007 Vital Health Stat 2011 13 1 38 21614897

[B2] Gupta K Hooton TM Naber KG Wullt B Colgan R Miller LG Moran GJ Nicolle LE Raz R Schaeffer AJ Soper DE International clinical practice guidelines for the treatment of acute uncomplicated cystitis and pyelonephritis in women: a 2010 update by the infectious diseases society of america and the European society for microbiology and infectious diseases Clin Infect Dis 2011 52 e103 120 10.1093/cid/ciq257 21292654

[B3] Gupta K Sahm DF Mayfield D Stamm WE Antimicrobial resistance among uropathogens that cause community-acquired urinary tract infections in women: a nationwide analysis Clin Infect Dis 2001 33 89 94 10.1086/320880 11389500

[B4] Karlowsky JA Kelly LJ Thornsberry C Jones ME Sahm DF Trends in antimicrobial resistance among urinary tract infection isolates of Escherichia coli from female outpatients in the United States Antimicrob Agents Chemother 2002 46 2540 2545 10.1128/AAC.46.8.2540-2545.2002 12121930 PMC127340

[B5] Hooton TM Johnson C Winter C Kuwamura L Rogers ME Roberts PL Stamm WE Single-dose and three-day regimens of ofloxacin versus trimethoprim-sulfamethoxazole for acute cystitis in women Antimicrob Agents Chemother 1991 35 1479 1483 10.1128/AAC.35.7.1479 1929311 PMC245194

[B6] European Association of Urology Guidelines on Urological Infections 2011 Arnhem, The Netherlands: http://www.uroweb.org/gls/pdf/15_Urological_Infections.pdf

[B7] Ulleryd P Zackrisson B Aus G Bergdahl S Hugosson J Sandberg T Prostatic involvement in men with febrile urinary tract infection as measured by serum prostate-specific antigen and transrectal ultrasonography BJU Int 1999 84 470 474 10468764 10.1046/j.1464-410x.1999.00164.x

[B8] Ulleryd P Febrile urinary tract infection in men Int J Antimicrob Agents 2003 22 Suppl 2 89 93 14527778 10.1016/s0924-8579(03)00228-0

[B9] Gupta K Hooton TM Stamm WE Increasing antimicrobial resistance and the management of uncomplicated community-acquired urinary tract infections Ann Intern Med 2001 135 41 50 11434731 10.7326/0003-4819-135-1-200107030-00012

[B10] Zhanel GG Hisanaga TL Laing NM DeCorby MR Nichol KA Weshnoweski B Johnson J Noreddin A Low DE Karlowsky JA Hoban DJ Antibiotic resistance in Escherichia coli outpatient urinary isolates: final results from the North American Urinary Tract Infection Collaborative Alliance (NAUTICA) Int J Antimicrob Agents 2006 27 468 475 10.1016/j.ijantimicag.2006.02.009 16713191

[B11] Lagace-Wiens PR Simner PJ Forward KR Tailor F Adam HJ Decorby M Karlowsky J Hoban DJ Zhanel GG Analysis of 3789 in- and outpatient Escherichia coli isolates from across Canada–results of the CANWARD 2007–2009 study Diagn Microbiol Infect Dis 2011 69 314 319 10.1016/j.diagmicrobio.2010.10.027 21353959

[B12] Hindler JF Barton M Callihan DR Erdman SM Evangelista AT Jenkins SG Johnston J Master R McGowan J John E Nimmo G Stelling J Analysis and Presentation of Cumulative Antimicrobial Susceptibility Test Data; Approved Guideline--Third Edition (M39-A3) Book Analysis and Presentation of Cumulative Antimicrobial Susceptibility Test Data; Approved Guideline--Third Edition (M39-A3) 2009 Wayne, Pennsylvania: Clinical and Laboratory Standards Institute 26

[B13] Linhares I Raposo T Rodrigues A Almeida A Frequency and antimicrobial resistance patterns of bacteria implicated in community urinary tract infections: a ten-year surveillance study (2000–2009) BMC Infect Dis 2013 13 19 10.1186/1471-2334-13-19 23327474 PMC3556060

[B14] Edlin RS Shapiro DJ Hersh AL Copp HL Antibiotic resistance patterns in outpatient pediatric urinary tract infections J Urol 2013 10.1016/j.juro.2013.01.069 PMC4165642 23369720

[B15] Zhanel GG Hisanaga TL Laing NM DeCorby MR Nichol KA Palatnik LP Johnson J Noreddin A Harding GK Nicolle LE Hoban DJ Antibiotic resistance in outpatient urinary isolates: final results from the North American Urinary Tract Infection Collaborative Alliance (NAUTICA) Int J Antimicrob Agents 2005 26 380 388 10.1016/j.ijantimicag.2005.08.003 16243229

[B16] Sobel JD Kaye D Mandell GL, Douglas RG, Bennett JE, Dolin R Urinary Tract Infections Mandell, Douglas, and Bennett's principles and practice of infectious diseases 2005 6 New York: Elsevier/Churchill Livingstone 875 905

[B17] McCue JD UTIs in at-risk patients: Are they 'complicated'? Infect Med 1999 16 533 540

[B18] Anderson RU Mulholland SG Treatment of complicated and uncomplicated urinary tract infections Antibiotic therapy in urology 1996 Philadelphia: Lippincott-Raven Publishers 23 37

[B19] Stuck AK Tauber MG Schabel M Lehmann T Suter H Muhlemann K Determinants of quinolone versus trimethoprim-sulfamethoxazole use for outpatient urinary tract infection Antimicrob Agents Chemother 2012 56 1359 1363 10.1128/AAC.05321-11 22232276 PMC3294920

